# Fatty acid challenge shifts cellular energy metabolism in a substrate-specific manner in primary bovine neonatal hepatocytes

**DOI:** 10.1038/s41598-023-41919-3

**Published:** 2023-09-12

**Authors:** T. L. Chandler, S. J. Kendall, H. M. White

**Affiliations:** 1 Department of Animal and Dairy Sciences, University of Wisconsin-Madison, Madison, WI 53706 USA; 2 College of Veterinary Medicine, Baker Institute for Animal Health, Cornell University, Ithaca, NY 14853 USA

**Keywords:** Biochemistry, Cell biology, Physiology

## Abstract

Adipose tissue mobilization increases circulating fatty acid (FA) concentrations, leads to increased hepatic FA uptake, and influences hepatic metabolism. Our objective was to trace carbon flux through metabolic pathways in primary bovine neonatal hepatocytes challenged with FA, and to examine the effect of FA challenge on oxidative stress. Primary bovine neonatal hepatocytes were isolated from 4 Holstein bull calves and maintained for 24 h before treatment with either 0 or 1 mM FA cocktail. After 21 h, either [1-^14^C]C16:0 or [2-^14^C]sodium pyruvate was added to measure complete and incomplete oxidation and cellular glycogen. Cellular and media triglyceride (TG), and glucose and ß-hydroxybutyrate (BHB) export were quantified, as well as reactive oxygen species and cellular glutathione (GSH/GSSH). Fatty acid treatment increased cellular, but not media TG, and although complete oxidation of [1-^14^C]C16:0 was not affected by FA, BHB export was increased. Reactive oxygen species were increased with FA treatment and GSSH was marginally increased such that the ratio of GSH:GSSG was marginally decreased. Glucose export increased, and cellular glycogen marginally increased with FA treatment while [2-^14^C]sodium pyruvate oxidation was decreased. These data suggest that FA treatment shifts cellular energy metabolism in a substrate-specific manner, spares pyruvate carbon from oxidation, and stimulates glucose synthesis.

## Introduction

In nonruminant species, the absorption of dietary glucose can support the body’s energy requirements and the liver modulates circulating glucose through uptake and utilization. In the fed state, nonruminants are predominately in a glycolytic state and can oxidize glucose for energy and once energy needs are met, subsequently synthesize glycogen or fatty acid (FA) for storage. In contrast, rumen fermentation of dietary carbohydrates minimizes the absorption of glucose in ruminant animals, and the animal spares glucose for obligatory glucose pathways rather than oxidizing it for energy. To support glucose needs, ruminants rely heavily on hepatic gluconeogenesis, especially during lactation when glucose requirements increase significantly to support milk lactose synthesis^1–3^. Gluconeogenesis is energetically expensive and fueled by ATP generated by tricarboxylic acid cycle (TCA) oxidation of FA or other oxidative precursors. These pathways are additionally linked through shared intermediates such as oxaloacetate and precursors that are both glucogenic and ketogenic such as propionate, lactate, and some amino acids.

Mobilization of triglycerides (TG) stored in the adipose tissue during negative energy balance is often associated with the onset of lactation and contributes glucose (e.g. glycerol) and oxidative (e.g., FA) precursors; however, unmatched supply of precursors and hepatic capacity can lead to metabolic disorders^4^. For example, excessive lipolysis is associated with ketosis and fatty liver in early lactation cows^5,6^, and hepatic lipidosis impaired metabolism^7,8^ and is associated with oxidative stress^9^. While there is substantial epidemiological work that supports a role of hepatic FA uptake in liver dysfunction, the direct effect of FA on hepatic carbon metabolism is not clear. Previous data suggested that FA accumulation in bovine hepatocytes decreased FA oxidation, gluconeogenesis, and ureagenesis^10–12^; however, other research demonstrated that individual FA increased gluconeogenesis^13,14^, and we have previously reported that FA increased gene expression of enzymes controlling FA metabolism and gluconeogenesis^15^. While the idiopathic accumulation of lipids in bovine hepatocytes likely alters cellular function, FA oxidation provides energy to fuel gluconeogenesis and other pathways and may also serve a role in stimulating gluconeogenesis.

We hypothesized that FA could alter the flux of oxidative and glucogenic carbon through pathways of energy metabolism in the liver. The primary objective of this experiment was to determine the effect of FA on carbon flux of palmitate and pyruvate through pathways of oxidation and gluconeogenesis in primary bovine neonatal hepatocytes. To accomplish this objective, pathway flux was determined by directly measuring pathway products and determining flux of [1-^14^C]palmitate and [2-^14^C] sodium pyruvate to labeled pathway products. Markers of oxidative balance were measured to determine changes in oxidative stress concurrently with cellular metabolism.

## Results

**Figure 1:**
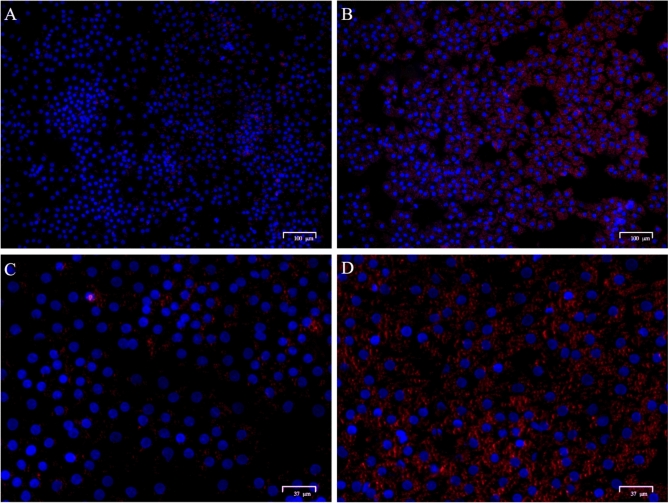
Primary bovine neonatal hepatocytes stained with DAPI (nuclei; blue) and Oil Red O (lipid droplet, red) to visually confirm hepatocyte lipid accumulation after 24 h of treatment with 0 mM (**A**,**C**) or 1 mM (**B**,**D**) fatty acid cocktail mimicking circulating FA profiles in bovine serum observed at calving. Images acquired with a ZOE microscope (BioRad). Scale bars edited for clarity.

**Figure 2:**
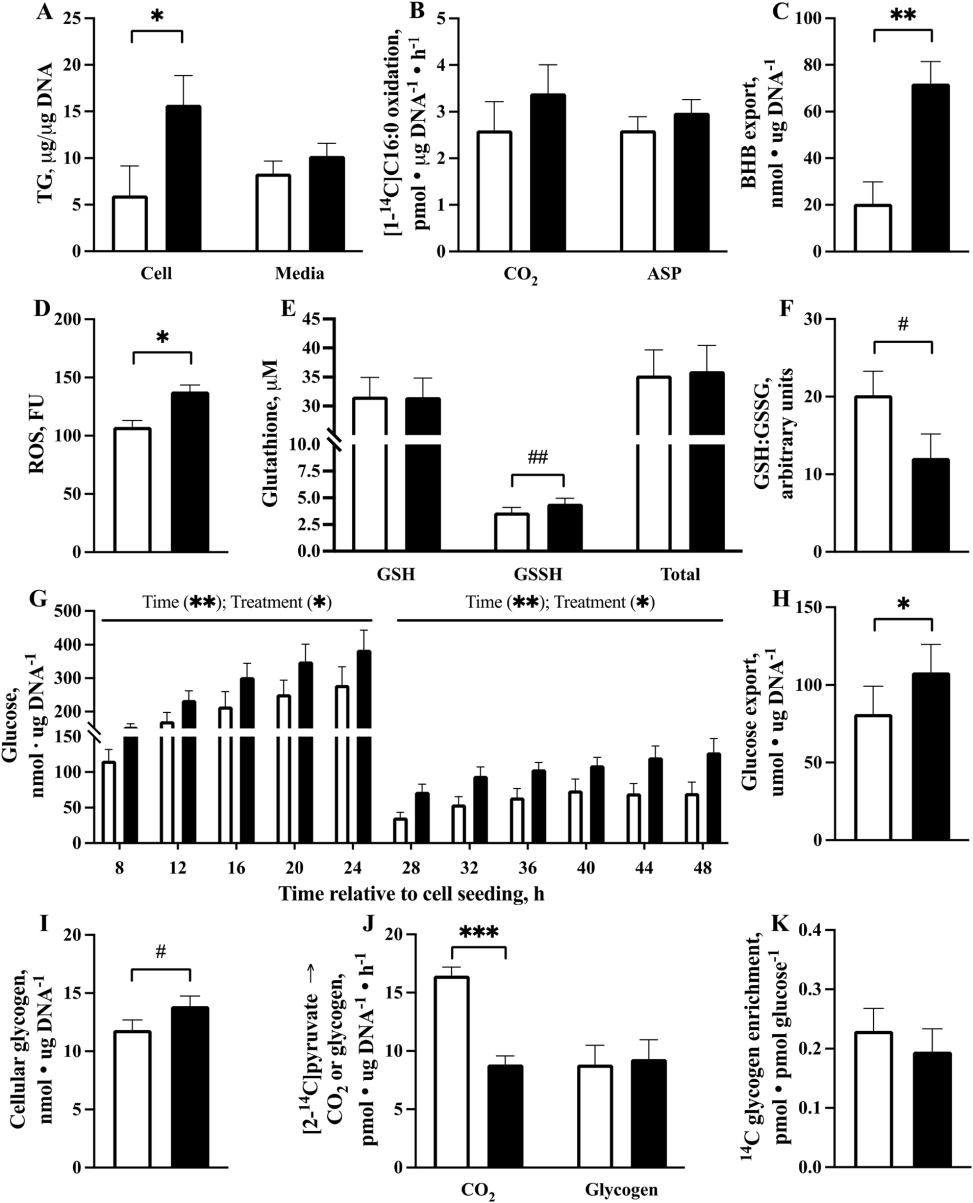
Changes in (**A**) cellular and media triglyceride (TG), (**B**) rate of [1-^14^C]C16:0 oxidation to CO_2_ or acid soluble products (ASP), (**C**) ß-hydroxybutyrate (BHB) export, (**D**) concentration of reactive oxygen species (ROS) in media, (**E**) cellular glutathione (GSH), cellular glutathione disulfide (GSSH), and total glutathione, (**F**) the ratio of GSH:GSSH, (**G**) media glucose concentration over time, (**H**) glucose export, (**I**) cellular glycogen, (**J**) rate of [2-^14^C] sodium pyruvate oxidation to CO_2_ and incorporation into glycogen, and (**K**) glycogen enrichment with ^14^C from [2-^14^C] sodium pyruvate in primary bovine neonatal hepatocytes treated with 0 (open bars) or 1 mM (closed bars) fatty acid (FA) cocktail. Cells were treated without or with a 1 mM FA cocktail for 24 h (**A**,**C**–**I**) or with a FA cocktail for 21 h before a 3 h incubation with [1-^14^C]C16:0 (**B**) or [2-^14^C] sodium pyruvate (**J**,**K**). (**G**) Media concentration of glucose for 48 h after cell seeding with media refreshed at 4 and 24 h after cell seeding; effect of time and treatment shown. Values are least squares means, with SE represented by vertical bars. *P* < 0.15 (#), *P* < 0.10 (##), *P* < 0.05 (*), *P* < 0.01 (**), *P* < 0.001 (***).

Visual assessment of cellular lipid accumulation was assessed using lipid droplet staining and representative images are shown in Fig. 1. The quantitative effect of FA treatment on cellular and media TG, metabolic rates of [1-^14^C]C16:0 metabolism, and ß-hydroxybutyrate (BHB) export is presented in Fig. 2A–C. Fatty acid treatment increased (*P* = 0.02) cellular TG but did not affect (*P* = 0.26) media TG. The rates of [1-^14^C]C16:0 complete oxidation to CO_2_ (*P* = 0.19) and incomplete oxidation to acid-soluble products (ASP; *P* = 0.30) were not affected by FA treatment. The export of BHB was increased (*P* = 0.008) by FA treatment.

The effect of FA treatment on reactive oxygen species (ROS) in media, cellular glutathione (GSH) and glutathione disulfide (GSSG), total glutathione, and the ratio of GSH:GSSG are presented in Fig. 2D–F. The accumulation of ROS in cell culture media was increased (*P* = 0.02) by FA treatment. Cellular GSH (*P* = 0.76) and total glutathione (*P* = 0.25) were not affected by FA treatment. Cellular GSSH increased with FA treatment (*P* = 0.07) and the ratio of GSH:GSSG was marginally decreased (*P* = 0.12) by FA treatment.

The concentration of glucose in media from 8 to 48 h of culture is presented in Fig. 2G. Glucose concentration increased (*P* < 0.001) during the first 24 h of culture and, after media was changed at 24 h, increased (*P* < 0.001) until 48 h of culture. Overall, fatty acid treatment increased (*P* = 0.02) glucose export from hepatocytes (Fig. 2H).

The effect of FA treatment on cellular glycogen and the metabolic rate of [2-^14^C] sodium pyruvate metabolism is presented in Fig. 2 (I–K). Cellular glycogen was marginally increased by FA treatment (*P* = 0.15). The rate of [2-^14^C] sodium pyruvate oxidation to CO_2_ decreased (*P* < 0.001) with FA treatment. The rate of [2-^14^C] sodium pyruvate incorporation into glycogen (*P* = 0.85) and glycogen enrichment with ^14^C from [2-^14^C] sodium pyruvate (*P* = 0.57) was not affected by FA treatment.

## Discussion

**Figure 3:**
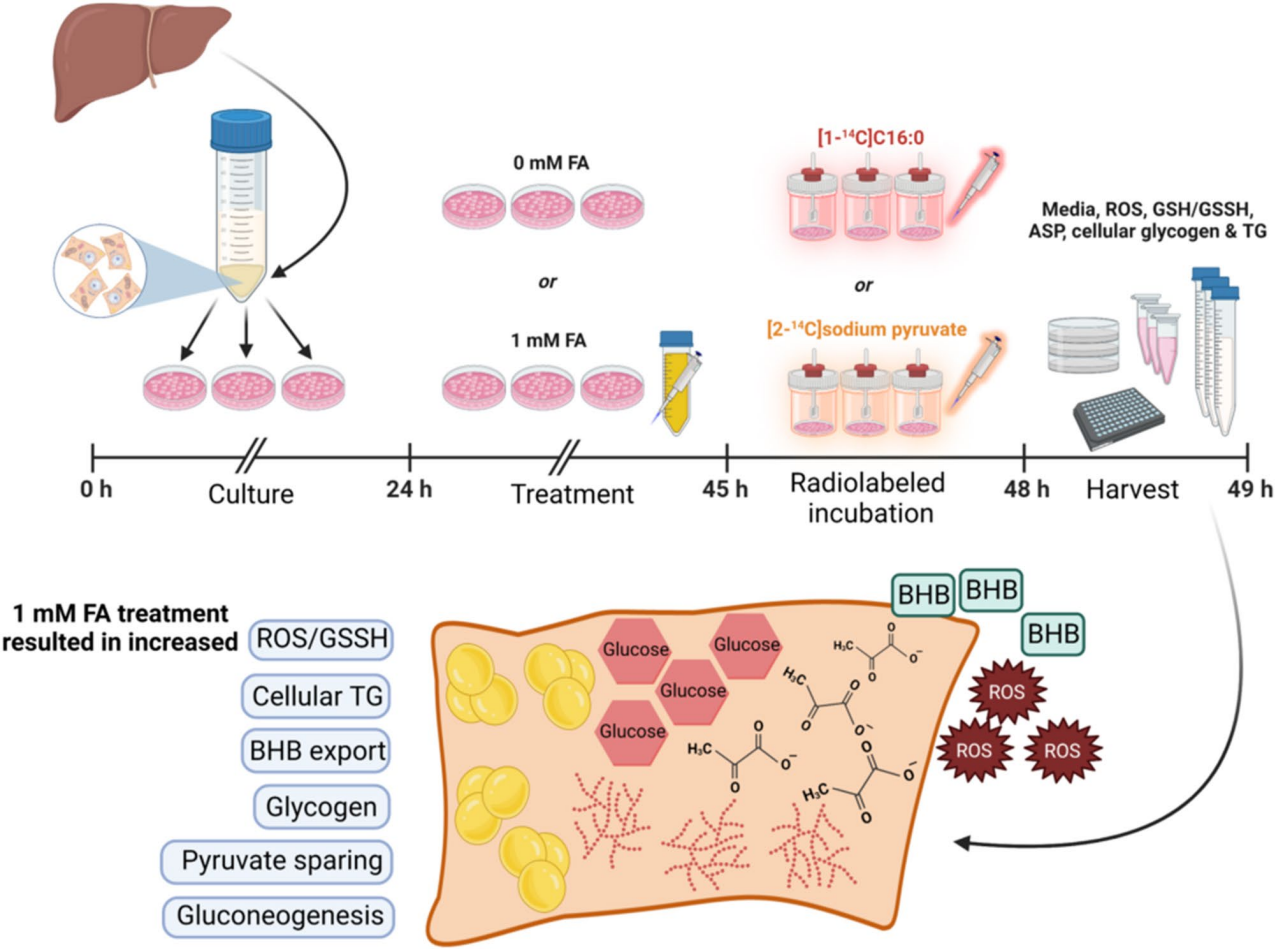
Schematic overview of experimental design and results in primary bovine neonatal hepatocytes treated with 0 or 1 mM fatty acid (FA) cocktail for 21 h before a 3 h incubation with [1-^14^C]C16:0 or [2-^14^C] sodium pyruvate. Reactive oxygen species (ROS), glutathione/glutathione disulfide (GSH/GSSH), acid soluble products (ASP), triglyceride (TG), and β-hydroxybutyrate (BHB).

Mobilization of stored TG during negative energy balance, especially postpartum, can present both gluconeogenic (e.g., glycerol) and oxidative (e.g., FA) precursors to the liver; however, FA uptake that exceeds oxidative capacity can lead to metabolic disorders^4^. Understanding the impact of FA exposure on gluconeogenesis and oxidative capacities can provide key information in understanding nutrient partitioning during states of negative energy balance. We previously reported that hepatocyte palmitate and pyruvate flux, in the presence of FA, are responsive to choline chloride and methionine (Met) supply in vitro^16^. In order to evaluate the effect of FA exposure on flux of pyruvate and palmitate in primary bovine neonatal hepatocytes, cells in the current experiment were treated with or without FA (Fig. 3). While choline chloride and Met were not of primary interest here, media was depleted of these nutrients for consistency. Given the short-term incubation, and presence of folic acid as a source of methyl-groups to support Met regeneration and choline synthesis, cell metabolism in this study was not likely influenced by lack of either aforementioned nutrient.

Use of a cell culture model, as done herein, allows for direct examination of pathway flux in response to changes in precursors. As the visual assessment confirms, hepatocytes in culture can take up media FA and subsequently store them as TG (Figs. 1, 2A). Our primary objective in the current experiment was to determine the effect of FA on carbon flux through pathways of oxidation and gluconeogenesis in primary bovine neonatal hepatocytes using a radiolabeled precursor of each pathway. While FA can provide a critical energy source for gluconeogenesis in the liver, hepatic uptake can exceed the capacity for FA oxidation and TG export as very-low density lipoprotein (VLDL), leading to lipidosis and ketogenesis^17^. Ruminants have a comparatively limited capacity to export TG as VLDL compared with nonruminants^18^. The increase in cellular TG, but not media TG, in hepatocytes challenged with FA in this study emphasizes the propensity for storage and accumulation, but not export, of FA taken up by bovine neonatal hepatocytes in this cell culture model. We have previously shown that primary cultures of bovine neonatal hepatocytes can export measurable TG in VLDL particles under similar in vitro conditions^16^. Despite the uptake and accumulation of FA supplied to hepatocytes, FA exposure did not increase [1-^14^C] palmitate complete and incomplete oxidation in the final 3 h of incubation which is in contrast to the cumulative increase in BHB export into media over the 24 h incubation period in the presence of FA which supports that incomplete oxidation was increased. This could be attributed to substrate availability and substrate driven regulation; however, previous work has demonstrated that gene expression of oxidative enzymes increased in response to FA treatment in a similar hepatocyte cell culture experiment^15^. Taken together, these data support that elevated FA uptake by hepatocytes can exceed capacity for complete oxidation via the TCA cycle and result in increased incomplete oxidation to ketone bodies and storage as TG.

These data confirmed that FA metabolism increased ROS accumulation and potentially oxidative stress from damaging radicals produced during mitochondrial oxidation^19^. It followed that GSSH was increased such that the ratio of GSH:GSSG was marginally decreased as GSH is oxidized to GSSG when scavenging ROS or free radicals^20^; however, we did not confirm if ROS generation and the corresponding change in redox buffering lead to the induction of inflammatory pathways and later steatosis. Because hepatocytes were pre-treated with FA before flux assay, we cannot delineate the effects of lipid accumulation or FA supply within the current experimental protocol, and it would take additional time course sampling to delineate when lipid accumulation and accompanied oxidative stress associated with lipid oxidation becomes idiopathic and cellular metabolism is subsequently altered. Of note, marginally increased cellular glycogen content, accompanying increased cellular TG, with FA treatment was unexpected in the present experiment as liver TG and glycogen are typically negatively correlated in vivo^21,22^, particularly in animals experiencing fatty liver^23^. It is possible that the storage of glycogen vs. release of glucose is different in vitro compared to in vivo and that glycogen deposition was favored under the conditions of this cell culture model. Hepatic lipidosis from in vivo sampling is often characterized on a wet or dry weight basis^24^, rather than normalized to DNA, and most reference thresholds are on a wet weight basis^6,24^. Despite this, it is valuable to note that the TG content in these monolayer cultures in the absence and presence of FA was consistent with hepatic TG on a DNA basis observed in prepartum, nonlactating cows with feed restriction^21^. Likewise, both prepartum and postpartum hepatic TG, DNA basis, in periparturient cows^25^ was similar to the cellular TG content of cells treated without or with FA in the current experiment. The consistency and physiological relevance of cellular TG content stored with and without challenge in vitro (e.g., incubation with FA) mimics what is observed in vivo (e.g., periparturient period or feed restriction) and highlights the relevance of the cell culture model used here.

Hepatic lipidosis has been credited for limiting hepatic metabolism and function^8,26^, and TG accumulation in bovine neonatal hepatocytes decreased FA oxidation, gluconeogenesis, and ureagenesis in vitro in some^10–12^, but not all experiments^13,14^. Our data does not support a detrimental effect of FA under the conditions of the present experiment given increased glucose export and marginally increased cellular glycogen with FA treatment. Increased glucose export from cells cultured with FA was realized within 4 h of exposure to FA, although we cannot determine if the source of glucose was gluconeogenesis or glycogenolysis during the first 24 h of culture with FA. As bovine liver lacks glucokinase activity required to sequester glucose intracellularly^27,28^, and we confirmed that glucose accumulated in media over the 48 h culture period, increased hepatocyte glucose and glycogen export at the end of the culture period likely reflects increased gluconeogenesis.

Transcriptional regulation of enzymes by FA in hepatocytes is a potential mechanism to increase gluconeogenesis and FA stimulation of gluconeogenesis has been characterized in nonruminants^29,30^. Hepatic energy metabolism is responsive to FA in vivo^31–33^, and individual FA appear to differentially regulate the expression of oxidative and glucogenic enzymes in Madin-Darby bovine kidney cells^34^. Fatty acids upregulated bovine *pyruvate carboxylase* (EC 6.4.1.1) promoter activity in H4IIE cells^35^, and we previously reported increased expression of oxidative and glucogenic enzymes in primary bovine neonatal hepatocytes treated with a similar FA cocktail used in the current experiment^15^. The increase in gluconeogenesis in this experiment is consistent with previous research indicating upregulation of oxidative and glucogenic enzymes with FA challenge in glucogenic bovine cells^15,34^ and supports a role of FA in the coordinated response to shifts in nutrient partitioning and utilization.

Although there are several gluconeogenic precursors available to bovine hepatocytes in vivo, some are also known to directly upregulate gene expression of gluconeogenic enzymes (e.g., propionate upregulation of *phosphoenolpyruvate carboxykinase* (EC 4.1.1.32))^36,37^. To avoid this confounder in the current experiment, pyruvate was used as an intermediate gluconeogenic precursor for tracing experiments. In addition, the contribution of glucogenic precursors that enter as pyruvate intermediates (e.g. lactate, glucogenic amino acids) is increased at the onset of lactation in dairy cows^1,3^ and pyruvate was used to represent important gluconeogenic carbon sources at this stage of lactation. The decrease in pyruvate oxidation in the presence of FA suggests that in the presence of other oxidative precursors, glucose precursors will be spared from oxidation. Despite the increase in cellular glycogen, we did not detect a difference in pyruvate incorporation into glycogen or glycogen enrichment with pyruvate carbon. It is possible that spared pyruvate carbon appeared in exported glucose at a greater rate than glycogen, but we could not distinguish these differential fates using the current methodology. Alternatively, spared pyruvate carbon may have increased the capacity of the TCA cycle for oxidation of other carbon sources, and could have contributed to increased FA oxidation given that pyruvate was present at the same concentration in all media, even when no labeled pyruvate was included.

In agreement with our hypothesis, exposure to FA altered carbon flux through metabolic pathways in primary bovine neonatal hepatocytes. We confirmed an increase in ketogenesis and ROS accumulation consistent with FA metabolism. Glucose export continued over the 48 h culture period, and increased glucose export and cellular glycogen with FA exposure observed were consistent with increased gluconeogenesis. These data suggest FA treatment shifts cellular nutrient use in a substrate-specific manner, spares pyruvate carbon from oxidation, and increases gluconeogenesis.

## Methods

All animal use and handling protocols were approved by the University of Wisconsin-Madison College of Agricultural and Life Sciences Animal Care and Use Committee. All experiments were performed in accordance with relevant guidelines and regulations and authors complied with the ARRIVE guidelines. Radioactivity work was conducted in accordance with the University of Wisconsin-Madison Environmental Health and Safety guidelines and regulations.

A detailed description of materials and methods has been previously reported^16^ and sample handling and preparation is briefly described here. The experimental design is also depicted visually in Fig. 3.

### Isolation and cell culture

Primary bovine neonatal hepatocytes were isolated from 4 Holstein bull calves < 7 d of age within 4 h of feeding by collagenase perfusion of the caudate process as previously described^38^. Isolated cells were plated on 35-mm Eppendorf culture plates (Eppendorf, Hauppauge, NY) in Dulbecco’s Modified Eagle’s Medium (DMEM, 2902, Sigma-Aldrich, St. Louis, MO) supplemented with 20% fetal bovine serum (FBS) and 1% antibiotic, antimycotic solution (A5955, Sigma-Aldrich, St. Louis, MO). Four h after initial plating, media was refreshed with a 10% FBS and 1% antibiotic, antimycotic DMEM media.

### Treatments

Monolayer cultures of cells were maintained for 24 h and were at least 80% confluent before exposure to treatments. Cells were randomly assigned to either a 0 or 1 mM FA treatment. To prepare the FA cocktail, individual stocks of FA C14:0, C16:0, C18:0, C18:1n-9 *cis*, C18:2n-6 *cis*, and C18:3n-3 *cis* (Sigma-Aldrich, St. Louis, MO) were bound to bovine serum albumin (BSA; Merck Millipore, Billerica, MA) by the method previously described^39^ and combined in molar ratios to create a stock FA cocktail that mimicked the profile of circulating FA observed in dairy cows at the time of calving^34^. The stock FA cocktail was comprised of 3% C14:0, 27% C16:0, 23% C18:0, 31% C18:1n-6 *cis*, 8% C18:2n-6 *cis*, and 8% C18:3n-3 *cis.* The stock FA cocktail was then added to cell culture media to achieve 1 mM concentration. Because glucose export was an outcome of interest, a glucose-free media was prepared in-house according to the formulation of DMEM 2902 (1.25 mM sodium pyruvate; Sigma-Aldrich, St. Louis, MO) without glucose and fortified with a vitamin mixture that was made according to the formulation of the commercially available Basal Medium Eagle (BME) vitamin solution (6891; Sigma-Aldrich, St. Louis, MO). To isolate the effects of FA treatment, Met and choline chloride were omitted from the DMEM formulation and vitamin solution, respectively, to prepare a Met- and choline chloride-free media given these methyl donor’s ability to affect hepatocyte energy metabolism as previously reported^15^. Culture media was the resulting glucose-, Met-, and choline chloride-free media containing 1% antibiotic, antimycotic with or without FA cocktail. The 0 mM treatment was supplemented with BSA to account for BSA supplied to cells in the FA cocktail.

All treatments were applied in triplicate and repeated in 4 independent preparations of cells. Within each cell preparation, treatments were replicated in 5 parallel incubations to allow for harvest of different outcomes: (1) reactive oxygen species and cellular glutathione, (2) cellular and media TG and export of glucose and BHB, (3) [1-^14^C] palmitate metabolism, (4) [2-^14^C] sodium pyruvate metabolism and cellular glycogen, and (5) serial sampling for glucose export.

### Reactive oxygen species and glutathione

After 24 h of treatment exposure, media was harvested from each plate and pooled within triplicate for immediate quantification of ROS by fluorometric assay (Hydrogen Peroxide Cell-Based assay, Cayman Chemical, Ann Arbor, MI). After removal of media, cell lysates were collected for subsequent quantification of glutathione (GSH) and glutathione disulfide (GSSG) by colorimetric assay (Glutathione Assay Kit, Cayman Chemical, Ann Arbor, MI) as previously described^16^. According to the manufacturer’s protocol, both GSH and GSSG were measured to reflect total glutathione. An aliquot of sample was used to derivatize GSH with 2-vinylpyridine before measuring GSSG separately. Measured GSSG was then subtracted from total glutathione to calculate GSH by difference.

### Cellular and media triglyceride, glucose and BHB export

After 24 h of FA treatment exposure, media was harvested from each plate, pooled within triplicate, and stored at −80 °C for subsequent quantification of glucose (Autokit Glucose, Wako Diagnostics, Richmond, VA), TG (L-Type Triglyceride M, Wako Diagnostics, Richmond, VA), and BHB (commercial kit, CataChemWell-T, Awareness Technologies, Westport, CT).

After removal of media, cells were harvested for subsequent quantification of cellular TG and DNA as previously described^16^. Total cellular TG was normalized to corresponding total DNA within each culture plate prior to averaging within triplicates. Total glucose, BHB, and TG in the pooled media sample were normalized to total DNA averaged across the triplicate.

To characterize the temporal pattern of glucose export in this culture system, a parallel set of isolated hepatocytes were cultured for the serial sampling of media for glucose analysis. After 4 h of incubation following cell seeding, media was refreshed with base media without glucose, Met, and choline chloride and cells were randomly assigned in triplicate to media containing either 0 or 1 mM FA. Cells were cultured for the next 20 h before cell media was refreshed with the same treatments. Over the culture period from 8 to 48 h, 25 µL of media from each triplicate was serially collected and pooled across triplicates every 4 h for subsequent analysis of glucose.

### Measuring metabolic flux

#### Palmitate oxidation

Following FA treatment exposure for 21 h, media was replaced with a treatment media that omitted the 1 mM FA cocktail and cells were incubated with [1-^14^C]C16:0 for 3 h as previously described^16^. Briefly, treatment media was removed, and each plate was placed into a straight-side wide-mouth 60 mL jar (Nalgene, Thermo Scientific, Waltham, MA). Treatment media was added to each plate and BSA-bound C16:0 containing [1-^14^C]C16:0 (Perkin Elmer, Waltham, MA) was added to achieve a final concentration of 1 mM C16:0 with approximately 1,000,000 disintegrations per minute (DPM) per plate. Media was gassed with 95% O_2_, 5% CO_2_ and jars were immediately sealed with lids modified to hold rubber stoppers (Kimble Chase, Vineland, NJ) fitted with a hanging plastic center well (Kimble Chase, Vineland, NJ) containing a sterile filter paper strip. Jars were incubated at 37° C for 3 h before CO_2_ and acid-soluble products (ASP), including ketone bodies, were collected as previously described^16,40^. Radioactivity in CO_2_ and ASP was determined by liquid scintillation counting. Cells were collected as previously described for the analysis of total DNA^16^.

The rate of complete oxidation of palmitate was determined for each plate and expressed as pmol ^14^C substrate metabolized to ^14^CO_2_·µg DNA^−1^·h^−1^ as described previously^16^. The rate of incomplete oxidation of palmitate for each plate was determined as the synthesis of ASP over time and expressed as pmol ^14^C substrate incorporated into ASP·µg DNA^−1^·h^−1^ as described previously^16^. The rate determined for each plate was averaged across each triplicate.

#### Pyruvate metabolism

Following treatment exposure for 21 h, media was replaced with a treatment media that omitted the 1 mM FA cocktail and cells were incubated in the presence of [2-^14^C] sodium pyruvate at approximately 275,000 DPM per plate for 3 h before CO_2_ was collected as described^16^. Cells were collected for subsequent isolation of total DNA and cellular glycogen was isolated by precipitation and converted to glucose units by acid-heat hydrolysis for quantification of glucose in cellular glycogen as previously described^16^. Radioactivity in CO_2_ and cellular glycogen after conversion to glucose units was determined by liquid scintillation counting.

The rate of pyruvate oxidation was determined for each plate and expressed as pmol ^14^C substrate metabolized to ^14^CO_2_·µg DNA^−1^·h^−1^. The rate of pyruvate incorporation into glycogen was determined for each plate and expressed as pmol ^14^C substrate recovered in glucose·µg DNA^−1^·h^−1^. The enrichment of pyruvate in glycogen was calculated for each plate as total pmol of ^14^C substrate recovered in glucose divided by total pmol of glucose in glycogen. Rate and enrichment determined for each plate was averaged across each triplicate.

### Statistical analysis

Data were analyzed by PROC MIXED of SAS 9.4 (SAS Institute Inc., Cary NC) in a model that accounted for fixed effects of FA treatment and the random effect of cell preparation. Glucose concentration in media during culture was analyzed for the first 24 h and then 28 to 48 h by repeated-measures ANOVA in a model that accounted for the fixed effect of hour and the random effect of cell preparation with a REPEATED statement for time, subject of calf, and compound symmetry covariance structure. Significance is declared at *P* ≤ 0.10 and tendencies at 0.10 < *P* ≤ 0.15. Data are reported as least-squares means and standard error of the mean.

## Data Availability

The data that support the findings of this study are available, upon reasonable request, from the corresponding author.
